# A novel role for Teneurin C-terminal Associated Peptide (TCAP) in the regulation of cardiac activity in the Sydney rock oyster, *Saccostrea glomerata*

**DOI:** 10.3389/fendo.2023.1020368

**Published:** 2023-02-06

**Authors:** Tomer Abramov, Saowaros Suwansa-ard, Patricia Mirella da Silva, Tianfang Wang, Michael Dove, Wayne O’Connor, Laura Parker, Fraser D. Russell, David A. Lovejoy, Scott F. Cummins, Abigail Elizur

**Affiliations:** ^1^ Centre for Bioinnovation, University of the Sunshine Coast, Maroochydore, QLD, Australia; ^2^ School of Science, Technology and Engineering, University of the Sunshine Coast, Sippy Downs, QLD, Australia; ^3^ Invertebrate Immunology and Pathology Laboratory, Department of Molecular Biology, Federal University of Paraíba, João Pessoa, Brazil; ^4^ New South Wales (NSW) Department of Primary Industries, Port Stephens Fisheries Institute Taylors Beach, Port Stephens NSW, Australia; ^5^ School of Biological, Earth and Environmental Sciences, The University of New South Wales, Kensington, NSW, Australia; ^6^ School of Health and Behavioural Sciences, University of the Sunshine Coast, Maroochydore, QLD, Australia; ^7^ Department of Cell and Systems Biology, University of Toronto, Toronto, ON, Canada

**Keywords:** teneurin, TCAP, stress, metabolism, heart, heart rate, transcriptome, oysters

## Abstract

Teneurin C-terminal associated peptide (TCAP) is an ancient bioactive peptide that is highly conserved in metazoans. TCAP administration reduces cellular and behavioural stress in vertebrate and urochordate models, yet despite numerous studies in higher animals, there is limited knowledge of its role in invertebrates. In particular, there are no studies on TCAP’s effects on the heart of any metazoan, which is a critical organ in the stress response. We used the Sydney rock oyster (SRO) as an invertebrate model to investigate a potential role for sroTCAP in regulating cardiac activity, including during stress. sroTCAP is localized to the neural innervation network of the SRO heart, and suggested binding with various heart proteins related to metabolism and stress, including SOD, GAPDH and metabotropic glutamate receptor. Intramuscular injection of sroTCAP (10 pmol) significantly altered the expression of heart genes that are known to regulate remodelling processes under different conditions, and modulated several gene families responsible for stress mitigation. sroTCAP (1 and 10 pmol) was shown to cause transient bradycardia (heart rate was reduced by up to 63% and for up to 40 min post-administration), indicative of an unstressed state. In summary, this study has established a role for a TCAP in the regulation of cardiac activity through modulation of physiological and molecular components associated with energy conservation, stress and adaptation. This represents a novel function for TCAP and may have implications for higher-order metazoans.

## 1 Introduction

An organism’s molecular and physiological response to stress is a crucial biological function determining its resilience and survival. A lack or imbalance in cellular stress-mitigating responses is known to promote cellular damage, disease, and mortality (1, 2), with different organs affected by, and responding to, stress differently (3). The heart, which supplies nutrients to tissues and cells, is vulnerable to stress. Various stress types are known to be deleterious to heart function and could lead to heart disease and failure (4–6). In invertebrates, heart rate is correlated with metabolic activity and can be used as a measure of an organism’s vigour and adaptation to different conditions (e.g. temperature, salinity, and feed) (7–10).

Oysters, especially intertidal species such as the Sydney rock oyster (SRO; *Saccostrea glomerata*), are continuously exposed to changing environments (e.g. temperature, salinity, pH, currents), pollutants (e.g. metals, dioxins, polychlorinated biphenyls), and pathogens, resulting in stress (11, 12). Stress is detrimental to oysters, resulting in inflammation, reactive oxygen species production, and impaired immune function (13–16). Consequently, the stress response could result in maladaptation, leading to immunosuppression, increased bacterial load, disease, and mortality (17–19). Several molecular and cellular factors are correlated with stress mitigation and resilience, one of which is teneurin and its peptide derivative, teneurin C-terminal associated peptide (TCAP). TCAP is a highly conserved bioactive peptide found in all metazoans, except the more basal phyla of Cnidaria, Ctenophora, Placozoa, and Porifera (20). The effects of TCAP have been investigated in murine models (rats and mice) (21–27), zebrafish (28) and a tunicate (29, 30). For molluscs, TCAP was first characterized in *S. glomerata*, including its potential functional activity (16).

Studies demonstrate a wide range of activities attributed to TCAP in animals. For example, it can modulate the behavioural stress response, reducing anxiety and addictive behaviour in rodents (21, 24, 25, 31). It has also been shown to protect fish larvae from cold-induced stress by raising their metabolic rate (28), increasing glucose uptake and ATP production in murine neurons, reducing serum glucose levels in an insulin-independent manner (27), and reducing the mitochondrial respiratory pathways in heat-stressed oysters (16). Furthermore, TCAP was shown to be neuroprotective in murine models, increasing cellular response to chemical stress, reducing necrosis, and increasing cell viability (26). TCAP increased neural pathfinding, axon growth and fasciculation of hippocampal and hypothalamic cells (32) and modulated the expression of brain-derived neurotrophic factor (BDNF) (23). Exposure of SRO to 5 pmol TCAP led to the modulation of the immune function (measured by phagocytosis) under stress and reduced the hemocyte reactive oxygen species abundance under ambient and stress conditions. Furthermore, TCAP modulated genes involved in multiple pathways, including immune genes, antioxidants, osmoregulation and mitochondrial complex, in stressed oysters. sroTCAP had binding affinity with superoxide dismutase (SOD) and GAPDH, where this interaction was linked to reduced apoptosis (16).

While the expression of teneurin in SRO was highest in the heart (16), the effects of TCAP on metazoan cardiac contractility and cardiac stress response is unknown. This study aimed to further our understanding of the mechanism of action and stress response of TCAP in invertebrates. For the first time, TCAP was studied in the heart using SRO as a molluscan model, where its effects on the physiology and molecular stress of the oyster were demonstrated.

## 2 Materials and methods

### 2.1 Experimental animals

Adult SROs used in this study were obtained from Port Stephens Fisheries Institute, NSW Department of Primary Industry and transferred to the University of the Sunshine Coast aquaculture lab, where they were housed in a closed recirculating tank containing natural seawater at 23°C ± 1°C and 34 ± 1 ppt and acclimated for 5-7 days before the experiments. Animals were routinely fed commercial microalgae concentrates (shellfish diet 1800^®^ and LPB, Reed Mariculture, USA) according to the manufacturer’s instructions.

Bivalve research does not require animal ethics approval in Queensland (Australia) under the animal ethics committee regulations, nonetheless, the animals were housed and handled with care to avoid unnecessary stress.

### 2.2 Immunohistochemistry detection of TCAP in SRO heart

Freshly excised whole SRO hearts from four adult animals were fixed in a 4% paraformaldehyde solution in phosphate-buffered saline (PBS) at 4°C overnight with gentle shaking. The fixed hearts were dehydrated with increasing concentrations of ethanol (30, 50, 70, 90, 95, and 100%) for 10 min in each solution. The dehydrated heart tissues were immersed in xylene twice for 15 min each before being embedded in paraffin three times (at 65°C for 40 min each). Paraffin infiltrated heart tissues were embedded in paraffin blocks, and tissue sections (8-10 µm thick) were subsequently prepared using a microtome (UM-MS355; ProSciTech), as previously done (33). Sections were deparaffinized using xylene and then hydrated using decreasing ethanol concentrations (100, 95, 90, 70, 50 and 30%) before washing twice with PBS. Sections were blocked with a blocking buffer (5% normal goat serum, 1% bovine serum albumin and 0.3% Triton-X in PBS) for 2 h at room temperature with gentle shaking. After three washes with PBS, the sections were incubated with primary antibodies, 1:1000: 1) anti-sroTCAP [custom produced: rabbit polyclonal (16)], and neural specific proteins 2) anti-gamma-aminobutyric acid (GABA) (CAT#20094, ImmunoStar, Hudson, WI, USA), 3) anti-Phe-Met-Arg-Phe-NH2 (FMRF-amide) (CAT# 20091, ImmunoStar, Hudson, WI, USA), and 4) anti-tyrosine hydroxylase (CAT# 22941, ImmunoStar, Hudson, WI, USA), in a blocking buffer at 4°C overnight with gentle shaking. For negative controls, sections were incubated with pre-adsorbed antibodies in blocking buffer as described above. Pre-adsorbed antibodies were made by incubating anti-sroTCAP antibodies (42 µg) with 210 µg of sroTCAP (1:5 ratio) at 4°C overnight in 10 ml blocking buffer before use. After three washes with PBS, secondary goat anti-rabbit antibodies (Alexa Flour 488 or Alexa Flour 568, 1:2000 dilution) were added and incubated at room temperature for 2 h with gentle shaking. After three PBS washes, the nuclei were stained with DAPI (1µg/ml) in PBS. Sections were imaged using a Leica DM5500 microscope and Leica DFC550 camera (Leica Microsystems).

### 2.3 Detection of sroTCAP-protein interaction using a pull-down assay

A pull-down assay using a commercially available kit was used to identify myocardial proteins that interact with sroTCAP, as per manufacturers instructions and as previously described (16). Briefly, biotinylated sroTCAP was bound to streptavidin gel beads (Pierce™ Biotinylated protein interaction pull-down kit; ThermoFisher Scientific, USA), washed with tris-buffered saline and blocked with a biotin solution. SRO heart lysate from 4 animals (250 µg total protein) in Pierce™ IP Lysis Buffer (ThermoFisher Scientific, USA) was incubated with sroTCAP bound beads for 2 h at 4°C with gentle shaking, then washed three times with acetate buffer (pH 5.0, NaCl 0.012 M) to remove unbound proteins. The bound proteins were recovered from the gel beads using 200µL elution buffer (pH 2.8) for 5 min. The elution solution was neutralized with 10 µL 1M Tris, and the elution was repeated once more. The pull-down assay was preformed twice. Tryptic peptides detected by LC-MS/MS using QTOF X500R mass spectrometer (AB SCIEX, Concord, Canada) from one replicate are presented in supplementary files (**Table S1**). Background protein binding in a negative control biotin-blocked gel beads without sroTCAP was subtracted from the final results (**Table S2**). The recovered proteins were trypsin digested in-gel before uHPLC tandem QTOF MS/MS analysis and peptide alignment to the SRO protein database as previously reported (34). The mass spectrometry proteomics data have been deposited to the ProteomeXchange Consortium *via* the PRIDE (35) partner repository with the dataset identifier PXD039155.

### 2.4 Transcriptomic evaluation of SRO heart stress treated following sroTCAP injection

Adult female SRO were used in this experiment to limit potential sex-related variations in gene expression. Before the experiment, the oyster’s shell edge was clipped using a bone cutter to expose the inner cavity, and then the oysters were allowed to recover for 5-7 days. The gap created access for injections (50 µL treatment) into the adductor muscle using a 27 G needle. Oysters were divided into 4 groups (n=6) (**Table 1**) (1): AS- oysters injected with filter-sterilized seawater (FSSW) and exposed to ambient conditions; (2) SS– oysters injected with FSSW and exposed to stress conditions; (3) AT- oysters injected with sroTCAP and exposed to ambient conditions; and (4) ST- oysters injected with sroTCAP and exposed to stress conditions. Following injections, oysters were rested outside the water for 10 min to allow the treatments to circulate. Oysters were then placed into 10 L tubs containing 4 L of seawater for 3 hours. The salinity and temperature of the natural seawater in the tubs were held at 34 ppt and 22°C [optimal condition range for SROs (36)] for ambient conditions and 15 ppt and 30°C to induce stress in SROs [stress conditions modified from Ertl and O’Connor (15)] (**Table 1**).

**Table 1:** Summary of oyster treatments (injection and incubation conditions) used in the heart transcriptome analysis (n=6 per group).

Group ID	Aim	Treatment injection	Salinity (ppt) and temperature (°C)
AS	Ambient condition- control	50 µL of FSSW	Normal salinity 34 ppt and 22 ± 1°C
SS	Stress condition- control	50 µL of FSSW	Low salinity (15 ppt) High temperature (30 ± 1°C)
AT	Determine TCAP effects at ambient conditions	5 pmol of sroTCAP in 50 µL FSSW	Normal salinity 34 ppt and 22 ± 1°C
ST	Determine TCAP effects at stress conditions	5 pmol of sroTCAP in 50 µL FSSW	Low salinity (15 ppt) High temperature (30 ± 1°C)

At the end of treatments, oysters were shucked, the pericardial membrane opened and the whole heart excised and blotted dry on clean Kimwipes (laboratory tissues) before being snap-frozen in liquid nitrogen and stored at -80°C. Two heart samples from each treatment were pooled (n=3 heart pairs/treatment) and processed for total RNA using a TRIzol reagent (ThermoFisher), according to the manufacturer’s protocol. Non-directional poly-A libraries were prepared using NEBNext^®^ Ultra RNA Library Prep Kit for Illumina^®^ and sequenced using NovaSeq 6000 PE150 by Novogene (Hong Kong), which generated over 18 million reads (~ 6 GB). Raw reads were deposited in the NCBI SRA database (accession PRJNA804582). The raw transcriptome data were processed using CLC genomic version 21 (Qiagen); QC check, trimming, mapping, and pair-wise differential gene expression (DEG) using default parameters except for mapping (against the SRO genome (37)), in which length fraction was changed to 0.5, and similarity fraction changed to 0.8. The list of DEGs of all comparisons is provided in **Table S3** and a list of the total gene counts, RPKM, TPM and CPM of each sample is provided in **Table S4**.

DEGs of each pair-wise comparison were filtered with a False Discovery Rate (FDR) value of ≤0.05 and log2 fold change of > 1 and < -1. The filtered DGEs were used to obtain the enriched gene ontology (GO) terms using Omics-box (BioBam, Spain 2020) with the default parameters. REVIGO was used to obtain the most representative GO terms from the enrichment list (38). A dispensability cut-off of < 0.1 was used to select the most representative GO terms. To visualize the enriched GO terms after filtering, the GO name, gene number, log size, and *P*-value were used in R Studio as previously described (39).

Additionally, a list of genes associated with stress; heat shock proteins (HSPs), antioxidants, apoptosis, metabolism and osmoregulation were retrieved from the literature (15, 40), and genes involved in heart function (41) were also investigated. *Teneurin* relative gene expression was evaluated by the reads per kilobase million (RPKM) between the four treatments and graphed.

### 2.5 Effect of synthetic sroTCAP delivery on SRO heart contractions

sroTCAP was administered to oysters *via* pericardial or intramuscular (IM) routes. For pericardial administration, SROs were shucked, and the pericardium membrane was cut to allow placement of a hook under the heart between the atrial and ventricular chambers and the hook was connected to a force transducer (ADInstruments), modified from Ha Park, Kim (42). The oysters were kept moist but not submerged in seawater to prevent the treatment solutions from spreading and acting on other tissues. Heart rate was recorded using LabChart 7 software (ADInstruments) at ambient temperature (22 ± 1°C). Baseline cardiac contractility was recorded after it stabilized (~2 min post heart connection). Once baseline measurements were determined, 50 µL of FSSW was administered into the pericardial cavity as a negative control, and the cardiac contractility was recorded for 5 min. Then, sroTCAP (1 and 10 pmol in 50 µL FSSW) was administered into the pericardial cavity, and the cardiac contractility was recorded for up to 2 h. Neurotransmitters serotonin (5-hydroxytryptamine; 5-HT) and acetylcholine (ACh) at 10 mM concentration were administered as positive controls to verify the integrity of the force transducer setup.

A separate experiment was performed to measure cardiac contractility after IM injection (using 27 G needle) of FSSW (negative controls) or 50 µL sroTCAP (10 pmol in FSSW). At least 24 h before the experiment, a small gap was created in the shell using a bone cutter to allow treatment injection directly into the adductor muscle. Following injections, oysters were kept outside the water for 10 min to allow sroTCAP/FSSW to circulate. Then, oysters were shucked, and the heart was connected to the force transducer as described above and continuously recorded for 2 h. Different animals were used for each treatment, therefore, no washing steps were required.

The contraction time of each beat (time between peak contractions) was measured. Ten to 20 contraction time-points were extracted for each treatment per animal (n=3-4) at ~20 min post sroTCAP/FSSW delivery and/or during the peak of the sroTCAP effect and HR was calculated as beats per minute (BPM).

### 2.6 Statistical analysis

All results are expressed as mean ± SEM. Statistical significance was assessed by paired t-test using SPSS software version 26 (IBM Corp, NY). *A priori* hypothesis of a-*P*-value < 0.05 was considered statistically significant.

## 3 Results

### 3.1 Detection and localization of TCAP in SRO heart sections

**Figure 1:**
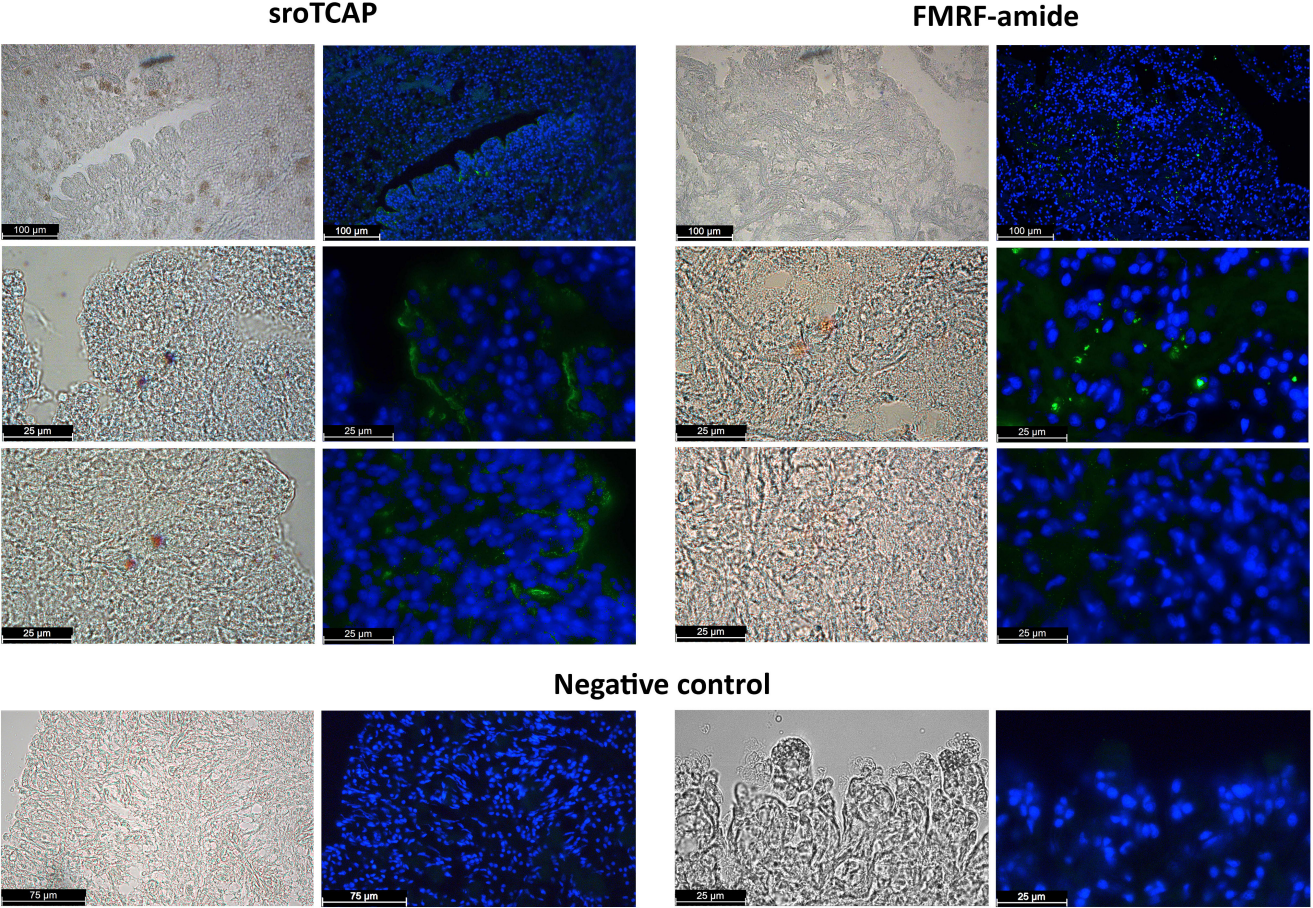
Immunohistochemistry of fixed SRO heart sections showing immunoreactivity using anti-TCAP antibodies (green) and anti-FMRF (green). Negative controls, preadsorbed antibodies. DAPI stain shows cell nucleus in blue. Scale bars 25, 75 and 100 µm.

TCAP immunoreactivity (ir-TCAP) was detected on the epithelial cell layer on the SRO heart periphery of both atrial chambers and to a lesser degree in the ventricle. In some regions of the atria (but not in the ventricle), cells with ir-TCAP were observed to penetrate from the peripheral epithelial tissue deeper into the heart connective tissue and muscle fibres (Atrium 1 and 2) (**Figure 1** and **Figure S1A**). Neuronal marker such as anti-GABA and anti-tyrosine hydroxylase showed a similar localization pattern on the peripheral epithelial tissue, and the immunoreactivity of anti-GABA anti-FMRF-amide was interspersed with cardiac muscle fibres and connective tissue (**Figure S1B**), also apparent in ir-TCAP.

### 3.2 Identification of heart proteins interacting with sroTCAP using a pull-down assay

Proteins interacting with sroTCAP were identified in a pull-down assay involving SRO heart protein lysate and biotinylated sroTCAP. Following mass spectrometry analysis, 28 unique proteins were identified (**Table 2**). These include several enzymes (e.g., carbonic anhydrase-like, putative aminopeptidase) and proteins involved in energy metabolism (e.g., cytosolic malate dehydrogenase, glyceraldehyde 3-phosphate dehydrogenase. Metabotropic glutamate receptor 1-like was the only receptor detected.

**Table 2:** Proteins identified from LC-MS/MS of pull-down assay using SRO heart lysate and biotinylated sroTCAP.

Gene ID	Protein annotation	-10lgP	Coverage (%)	#Peptides	#Unique
Sgl023942	extracellular superoxide dismutase [Cu-Zn]-like	183.32	22	16	14
Sgl004322	carbonic anhydrase-like	157.18	23	6	6
Sgl023943	extracellular superoxide dismutase [Cu-Zn]-like*	131.79	18	8	6
Sgl013490	complement C1q tumor necrosis factor-related protein 3	128.36	38	5	5
Sgl018613	heat shock protein 20	105.18	39	5	5
Sgl018612	major egg antigen-like	97.48	19	3	3
Sgl001060	septin-11-like isoform X2	93.80	10	4	3
Sgl016864	septin-2 isoform X5	93.15	9	3	3
Sgl009849	AAAChain AAA, Streptavidin	76.06	22	2	2
Sgl003905	glyceraldehyde 3-phosphate dehydrogenase*	71.78	8	2	2
Sgl018292	cytosolic malate dehydrogenase	64.65	6	1	1
Sgl003210	putative aminopeptidase W07G4.4	55.04	4	1	1
Sgl003464	cysteine and glycine-rich protein	52.17	9	1	1
Sgl001248	ependymin-related protein 1	50.84	18	1	1
Sgl025885	arginine kinase	44.93	3	1	1
Sgl024699	citrate synthase, mitochondrial-like	44.14	4	2	1
Sgl009737	isocitrate dehydrogenase [NAD] subunit alpha, mitochondrial-like	43.57	3	1	1
Sgl009738	isocitrate dehydrogenase [NAD] subunit alpha, mitochondrial-like	43.57	5	1	1
Sgl001979	RNA-binding protein FUS isoform X6	38.20	16	1	1
Sgl009446	complement C1q-like protein 4	33.14	4	1	1
Sgl003271	heat shock protein 20	31.93	3	1	1
Sgl005741	septin-7 isoform X1	31.36	2	1	1
Sgl012664	transitional endoplasmic reticulum ATPase	30.88	1	1	1
Sgl009430	small nuclear ribonucleoprotein G-like	29.40	18	1	1
Sgl019656	WASH complex subunit 2C-like isoform X3	26.43	1	2	1
Sgl005970	protein crumbs-like isoform X1	26.37	0	2	1
Sgl025823	metabotropic glutamate receptor 1-like	23.27	2	1	1
Sgl020233	fucolectin-1	21.33	6	1	1

-10lgP value represents the confidence of protein identification (cut-off value >20), Coverage represents the detected fragment coverage of the full protein, #Peptides represent the total number of peptide hits per protein, and #Unique represents the number of unique peptides detected per protein. *, also identified in Abramov, Suwansa-ard (16).

### 3.3 The effects of sroTCAP on gene expression in SRO heart under ambient and stress conditions

**Figure 2:**
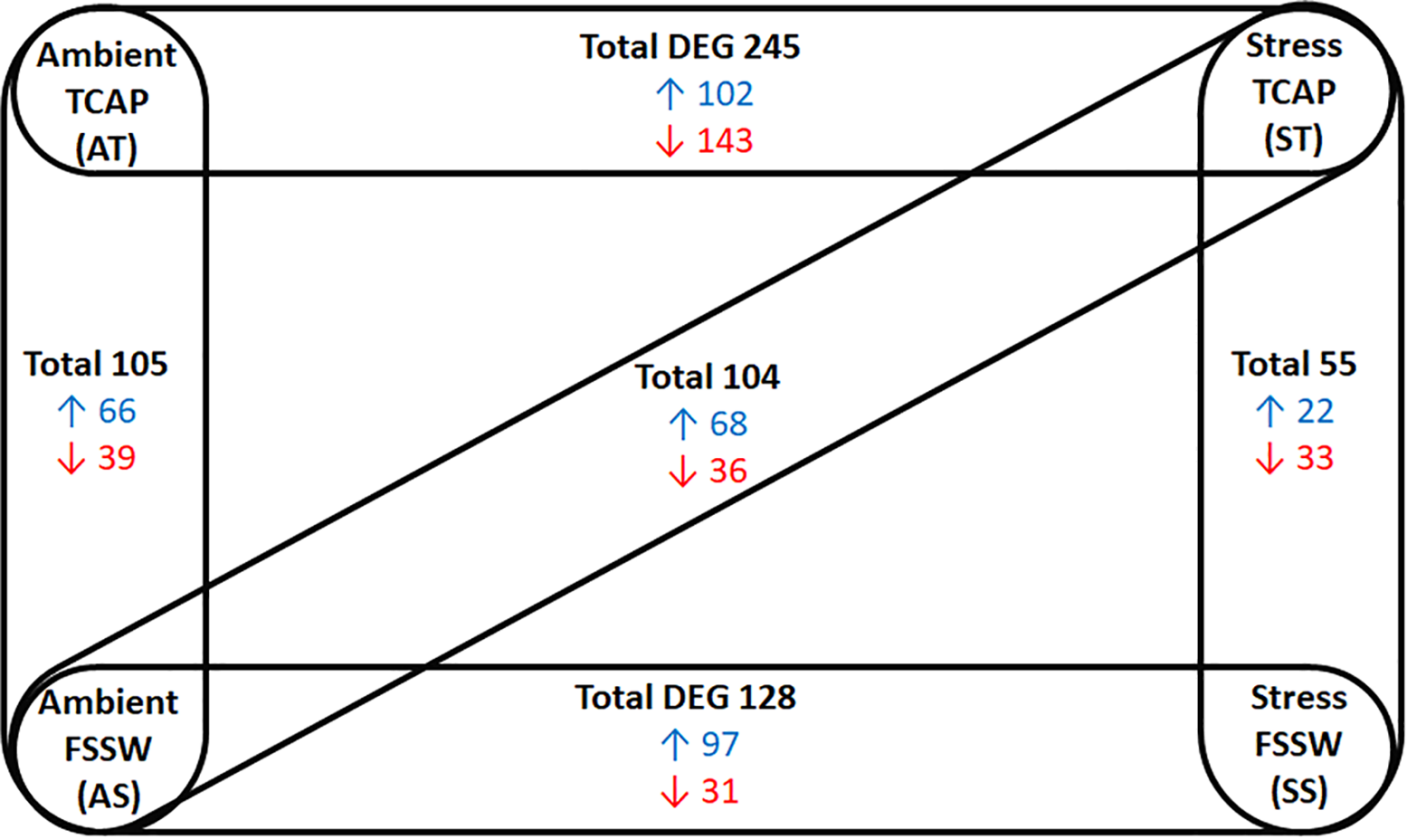
Changes in gene expression in SRO heart in response to sroTCAP or FSSW exposed to either ambient or stress conditions, showing five different pair-wise comparisons. Experimental groups showed significant DEG (at FDR ≤ 0.05 with log fold change ≥ 1 and ≤ -1) in each pair-wise comparison. Blue: upregulated DEG, red: Down-regulated DEG.

To assess the impact of sroTCAP on the heart under stress conditions, the oysters were injected with sroTCAP or FSSW (control) before being exposed to either ambient or stress (low salinity and high temperature) conditions (**Table 1**). Quantitative heart transcriptomes derived from four treatments were evaluated using five pair-wise comparisons (**Figure 2**). The DEGs in ST vs AT represent the effects of stress under sroTACP injection, SS vs AS represent the effects of stress under seawater injection and both-effected DEGs are represented by ST vs AS. The effects of sroTCAP between ambient and stress conditions is evaluated by comparing DEGs between AT vs AS and ST vs SS, respectively. A comparison of the stress controls (FSSW injected, SS *vs* AS) identified a total of 128 DEGs (97 upregulated and 31 downregulated). A comparison of ST *vs* SS (stress with and without sroTCAP administration) identified that sroTCAP had the lowest number of differentially expressed genes, 22 upregulated and 33 downregulated genes. The highest number of DEGs was observed for ST *vs* AT, where 245 genes were differentially expressed, 102 upregulated and 143 downregulated.

**Figure 3:**
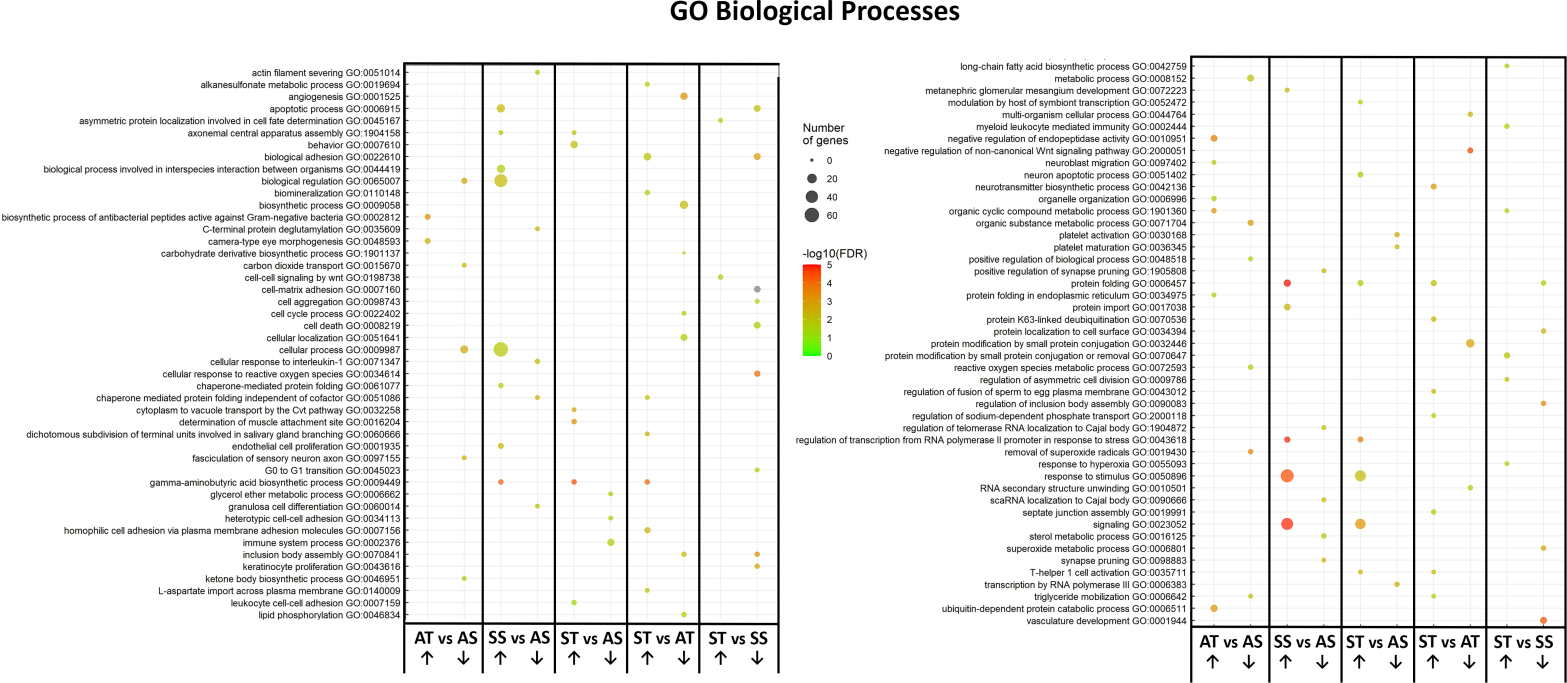
Changes in heart GO term profile in response to TCAP or FSSW under ambient or stress treatments. The plots show the over-represented and most significant biological processes in the upregulated and downregulated DEG list in response to the treatments. The dot size indicates the number of DEGs associated with the process, and the dot colour indicates the significance of the enrichment [-log10 (FDR-corrected *P*-values)]. Upward-pointing arrows represent the upregulated GO terms, and the downward-pointing arrows represent the downregulated GO terms.

Enriched GO annotation analysis between the comparisons found 19 terms in AT *vs* AS and ST *vs* AS, 20 in ST *vs* SS and 24 in SS *vs* AS. Comparison of ST *vs* AT had 27 GO terms, the highest number among the comparison groups. Several GO terms were found both in stressed SRO with and without sroTCAP, which could be considered stress-specific gene changes. These included ‘axonemal central apparatus assembly’, ‘gamma-aminobutyric acid biosynthetic processes’, ‘response to stimuli’, ‘signalling’, ‘protein folding’, and ‘regulation of transcription from RNA polymerase II promoter’ in response to stress. ‘Triglyceride mobilization’ was downregulated with sroTCAP at ambient but upregulated with sroTCAP and stress (**Figure 3**).

Some GO terms were sroTCAP specific, including ‘apoptotic process’, which was upregulated in the stress control (SS *vs* AS) but downregulated under stress with sroTCAP administration (ST *vs* SS). ‘Reactive oxygen species metabolic process’ was also downregulated when sroTCAP was delivered at ambient compared to ambient control (AT *vs* AS). ‘T-helper 1 cell activation’ was upregulated in stress sroTCAP compared to FSSW at ambient (ST *vs* AS) and sroTCAP at ambient (ST *vs* AT). ‘Inclusion body assembly’ was downregulated in sroTCAP stress compared to sroTCAP at ambient (ST *vs* AT) and stress FSSW (ST *vs* SS) (**Figure 3**).

Following a search for genes of interest related to heart function, stress, and environmental adaptation, their DEG was evaluated in all comparisons (**Table 3**). Two different isoforms of *extracellular superoxide dismutase [Cu-Zn]-like* were downregulated in sroTCAP at ambient (AT *vs* AS) and stress sroTCAP (ST *vs* SS) and were not differentially expressed in the other comparisons. Three downregulated *cytochrome P450* isoforms were found only in AT *vs* AS, and a single isoform was upregulated in ST *vs* AT. *NADPH oxidase 5-like isoform X2* was upregulated in both stress control (SS *vs* AS) and in ST *vs* AT and is therefore considered stress-specific.

**Table 3:** The analysis of the differentially expressed genes (DEGs) related to the cellular response to stress among the treatment groups.

Gene ID	Gene name	AT*vs*AS	SS*vs*AS	ST*vs*AS	ST*vs*AT	ST*vs*SS
Antioxidants and respiration						
Sgl018668	alcohol dehydrogenase [acceptor] isoform X2	X	X	-2.88	X	X
Sgl018679	alcohol dehydrogenase [acceptor]	X	X	X	3.394	X
Sgl023943	extracellular superoxide dismutase [Cu-Zn]-like	-1.88	X	X	X	-1.76
Sgl023942	extracellular superoxide dismutase [Cu-Zn]-like	-1.60	X	X	X	-1.81
Sgl017373	cytochrome P450 3A28	X	X	X	1.41	X
Sgl017374	cytochrome P450 3A28	-1.41	X	X	X	X
Sgl003130	cytochrome P450 2B15	-3.18	X	X	X	X
Sgl015406	cytochrome P450 3A9	-1.86	X	X	X	X
Sgl020530	NADPH oxidase 5-like isoform X2	X	1.51	X	1.41	X
Heat shock protein						
Sgl015508	heat shock 70 kDa protein 12A-like	X	X	-5.18	-4.82	X
Sgl010885	major heat shock 70 kDa protein Ba-like	X	6.00	4.97	4.05	X
Sgl011686	Heat shock protein beta-1	X	3.81	3.55	X	X
Sgl002779	heat shock protein 70 B2-like	X	4.84	3.30	3.54	X
Sgl002778	heat shock protein 70 B2-like	X	3.25	2.17	1.86	X
Sgl026767	heat shock 70 kDa protein 12A-like	-7.48	X	X	X	X
Sgl021413	heat shock 70 kDa protein 12A-like	2.24	X	X	X	X
Sgl021425	heat shock 70 kDa protein 12A-like	X	X	X	-2.11	X
Sgl020234	heat shock 70 kDa protein 12A-like	X	X	X	2.60	X
Sgl002777	heat shock protein 70 B2-like	X	3.14	X	X	X
Sgl018613	heat shock protein 20	X	1.96	X	X	X
Sgl002817	heat shock protein 70 B2-like	X	1.82	X	X	X
Sgl010884	heat shock protein 68-like	X	1.56	X	X	X
Apoptosis						
Sgl026039	baculoviral IAP repeat-containing protein 2-like	2.76	2.51	X	X	X
Sgl027379	baculoviral IAP repeat-containing protein 2-like	2.76	2.94	X	X	X
Sgl007395	apoptosis 1 inhibitor	X	1.96	X	X	X
Sgl026043	putative inhibitor of apoptosis	X	-3.54	X	X	X
Sgl009101	putative inhibitor of apoptosis	2.36	X	X	X	X
Sgl019716	death domain-containing protein 1-like	X	3.44	X	X	X
cardiac structural proteins		X	X	X	X	X
Sgl029591	myosin-16-like isoform X4	X	X	-3.24	-3.35	- 2.89
Sgl007355	myosin heavy chain, striated muscle isoform X1	-1.77	X	X	X	X
Sgl018625	paramyosin-like isoform X3	X	-2.50	-2.96	-2.76	X
Transcription regulators						
Sgl022632	zinc finger BED domain-containing protein 1-like	-2.65	X	X	X	X
Sgl028652	putative zinc finger protein	X	3.88	X	X	-5.91
Sgl010679	helicase with zinc finger domain 2-like	X	X	X	-1.45	
Sgl024879	zinc finger CCCH domain-containing protein 13 isoform X1	X	X	2.70	X	X
Sgl025509	NFX1-type zinc finger-containing protein 1-like	X	X	1.60	X	X
Sgl007933	probable transcription-associated protein 1	X	X	X	4.57	X
Sgl011986	cotranscriptional regulator FAM172A isoform X2	X	X	X	-1.52	X
Sgl007933	probable transcription-associated protein 1	X	X	X	X	6.01
Osmoregulation and ion transporters						
Sgl001382	excitatory amino acid transporter 1 isoform X3	X	2.60	2.08	1.63	X
Sgl020213	Zinc transporter 5	X	X	2.17	1.97	X
Sgl017400	iron transport multicopper oxidase fetC isoform X1	X	X	X	-1.86	X
Sgl003681	solute carrier family 23 member 2-like	X	1.29	X	X	X
Sgl018776	solute carrier family 28 member 3-like	X	X	X	3.55	X
Sgl011440	electroneutral sodium bicarbonate exchanger 1-like	4.76	X	X	X	X
Sgl006020	sodium- and chloride-dependent neutral and basic amino acid transporter B(0+)-like isoform X2	X	X	3.83	X	X
Sgl025271	proton channel OtopLc-like isoform X10	X	X	5.35	X	X
Sgl006757	voltage-dependent calcium channel subunit alpha-2/delta-3-like	X	X	X	-2.07	X
Intracellular Ca^2+^ channel						
Sgl011165	inositol 1,4,5-trisphosphate receptor (IP3R)	X	X	X	-1.64	X

Of the 13 differentially expressed HSPs detected, 8 were upregulated in the stress control group (SS *vs* AS). However, two upregulated and one downregulated *heat shock 70 kDa protein 12A-like* isoforms were specific to ST groups, while in the sroTCAP ambient control group (AT *vs* AS), one isoform was up and downregulated.

Apoptosis-related genes were investigated and were differentially expressed only in stress control (SS *vs* AS) and ambient sroTCAP control (AT *vs* AS) (**Table 3**). Two *baculoviral IAP repeat-containing protein 2-like* isoforms were similarly upregulated in stress control and ambient sroTCAP control. A single isoform of *putative inhibitor of apoptosis* was upregulated in sroTCAP ambient control and downregulated in the stress control group. A *death domain-containing protein 1-like* gene was upregulated in the stress control group.

Genes encoding structural heart proteins were evaluated and *myosin-16- like isoform X4* was downregulated in all ST comparisons, while *myosin heavy chain, striated muscle isoform X1* was downregulated in AT *vs* AS (**Table 3**). *Paramyosin-like isoform X3* was downregulated in the stress control (SS *vs* AS) and in two ST comparisons therefore, the changes are considered stress-specific and unrelated to sroTCAP.

Transcription regulators were evaluated to assess their differences among the different groups (**Table 3**). Of the eight differentially expressed transcription regulators detected, only a single gene (*putative zinc finger protein*) was found in the stress control group (SS *vs* AS). *Zinc finger BED domain-containing protein 1-like* was downregulated in ambient sroTCAP control (AT *vs* AS) while the rest were found in ST comparisons. Of those, *zinc finger CCCH domain-containing protein 13 isoform X1*, *NFX1-type zinc finger-containing protein 1-like* and *probable transcription-associated protein 1* were upregulated, while *putative zinc finger protein*, *helicase with zinc finger domain 2-like* and *cotranscriptional regulator FAM172A isoform X2* were downregulated.

Transporters and osmoregulation-related genes were evaluated to assess the heart’s response to the low salinity stress (**Table 3**). *Excitatory amino acid transporter 1 isoform X3* and *solute carrier family 23 member 2-like* were both found in the stress control (SS *vs* AS) and are considered stress-related. *Zinc transporter 5*, *solute carrier family 28 member 3-like, sodium- and chloride-dependent neutral and basic amino acid transporter B(0+)-like isoform X2* and *proton channel OtopLc-like isoform X10* were upregulated in at least one comparison having an ST group, while *iron transport multicopper oxidase fetC isoform X1* and *voltage-dependent calcium channel subunit alpha-2/delta-3-like* were downregulated in ST *vs* AT.

Membrane proteins involved in calcium influx in heart tissue were investigated, and only *inositol 1,4,5-trisphosphate receptor (IP3R)* was differentially expressed and found to be downregulated in ST *vs* AT comparison (**Table 3**). However, the following genes were not differentially expressed in any of the comparisons; *ryanodine receptor (RYR)*, *L-type Ca2+ channel* and *stromal interaction molecule 1 (STIM1)*.

**Figure 4:**
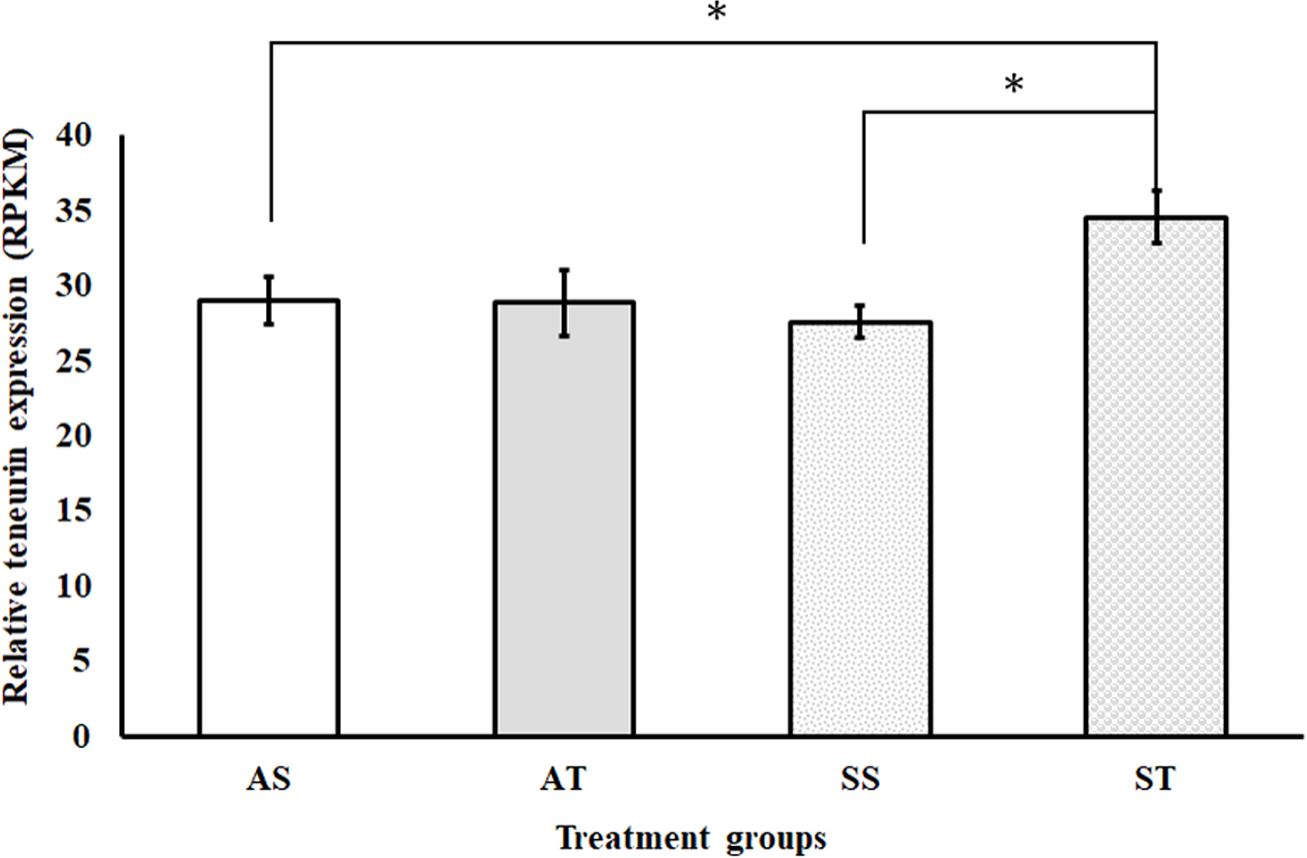
Relative expression (RPKM) of the *teneurin* gene in the heart of SRO under ambient or stress conditions and IM-injected with 5 pmol sroTCAP or FSSW (n=3). AS- oysters at ambient condition injected with FSSW, AT- oysters at ambient condition injected with sroTCAP, SS- oysters under stress condition injected with FSSW and ST- oysters under stress condition injected with sroTCAP. *P ≤ 0.05.

Meanwhile, sroTCAP delivery to oysters maintained under stress conditions increased *teneurin* transcript expression compared to ambient and stress controls (**Figure 4**). The expression of *teneurin* significantly increased when sroTCAP was delivered to oysters under stress (ST) compared to oysters under stress administered with FSSW (SS) (*P =* 0.038). Additionally, ST group had a significant increase in *teneurin* expression compared to the ambient control (AS) (*P =* 0.0167).

### 3.4 Physiological effects of sroTCAP delivery to SRO heart

**Figure 5:**
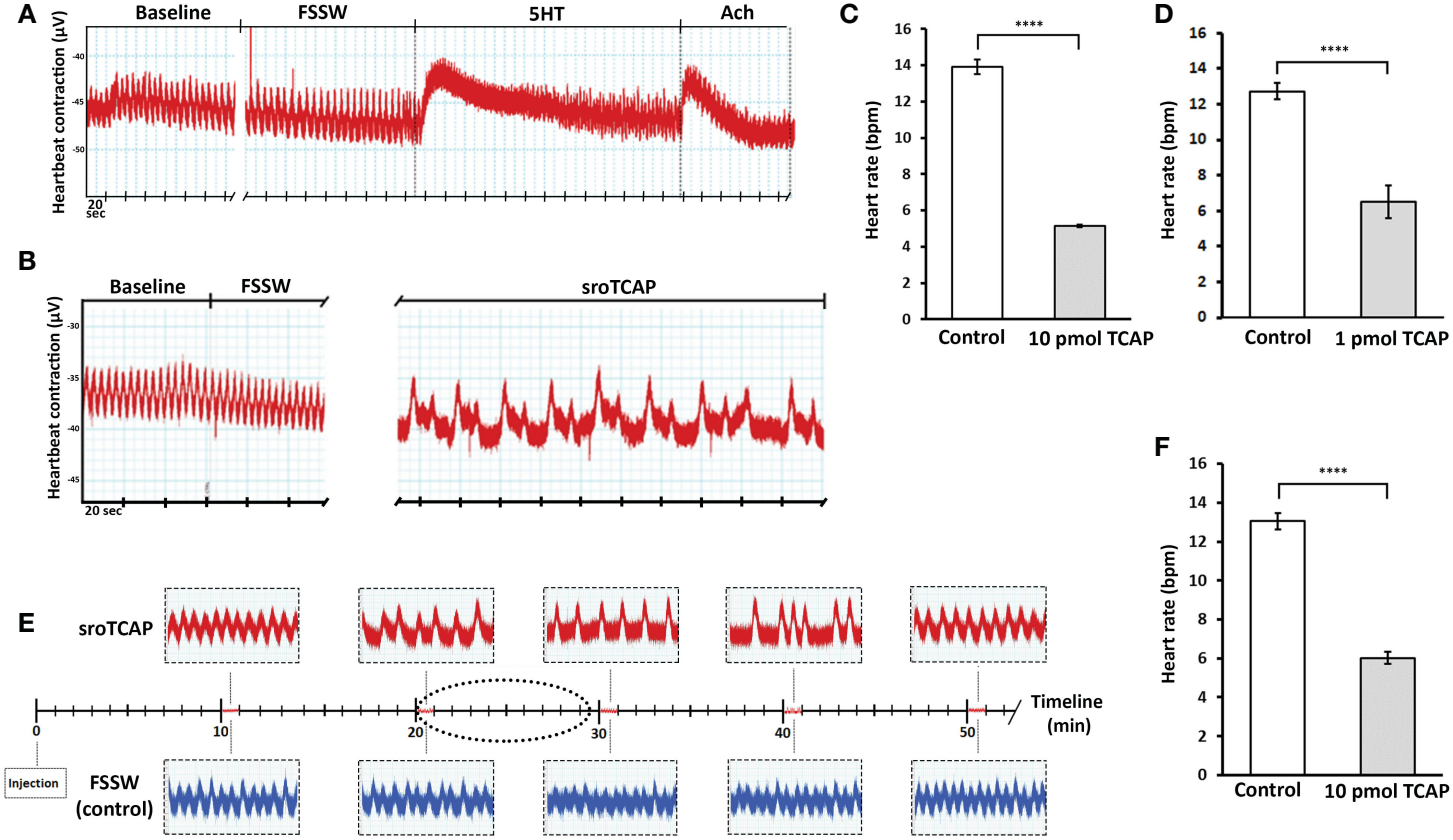
SRO heart rate following pericardial and IM administration of sroTCAP, FSSW and neurotransmitters. **(A)** Representative trace showing baseline heart rate following FSSW, 10 mM serotonin (5-HT) and 10 mM acetylcholine (ACh). **(B)** Representative trace showing baseline heart rate following FSSW and 10 pmol sroTCAP. **(C)** Histogram showing the frequency change in oyster heart rate (beats per min; BPM) in response to FSSW (control) and 10 pmol sroTCAP (n=3). **(D)** Histogram showing the frequency change in oyster heart rate (BPM) in response to FSSW (control) (n=3) and 1 pmol sroTCAP (n=4). **(E)** Heart rate comparison between TCAP and FSSW injected oysters showing 1 min timeline of heart rate traces at different time-points post-TCAP injection IM (red) and FSSW injection IM (blue). The dotted ellipse shows the region of data sampling. **(F)** Histogram showing the frequency change of BPM in FSSW control and TCAP IM-injected groups (n=4), (****P < 0.0001).

In order to examine the direct effect of sroTCAP on the heart, SRO heart tissue was treated with sroTCAP *via* the pericardial cavity, and changes in HR were analyzed. No change in heart rate was observed when FSSW was applied, while the positive controls, 5-HT and ACh (10 mM) caused a short spike in the amplitude of heart contraction and increased HR (**Figure 5A**). The effects lasted approximately 2.5 and 1 min for 5-HT and Ach, respectively, before returning to near baseline levels. In response to pericardial sroTCAP (10 pmol) delivery, changes in heart rate was observed at approximately 2 min post-administration, showing the onset of bradycardia (**Figure 5B**). At 10 pmol sroTCAP, the oyster heartbeat reduced significantly (*P* < 0.0001 (**Figure 5C**; **Table S5**). A 10-fold lower sroTCAP dose, (1 pmol), had also significantly (*P*<0.0001) reduced the HR, inducing bradycardia (**Figure 5D**; **Table S5**).

The administration of sroTCAP (10 pmol) by IM injection caused bradycardia 15 min post-injection and continuing until ~40 min post-injection, after which arrhythmia occurred (**Figure 5E**). By ~50 min post-injection, HR returned to baseline level with sinus rhythm. During the peak of bradycardia, HR slowed significantly (*P*<0.0001) (**Figure 5F**; **Table S5**). In addition to causing transient bradycardia, sroTCAP also increased the amplitude of the heart contractions (not quantified).

## 4 Discussion

TCAP is a bioactive neuropeptide that modulates the molecular, behavioural and metabolic stress responses in vertebrates and urochordates (22, 25, 28). Recently, Abramov, Suwansa-ard (16) demonstrated that sroTCAP administration in stressed oysters altered phagocytosis, ROS production and hemocyte gene expression relative to controls. Although *teneurin* orthologues were known to be expressed in the developing hearts of vertebrates (i.e., *Gallus gallus*) and invertebrates (i.e., *Drosophila*) (43, 44), the effect of TCAP administration on cardiac contractility and role in cardiac activity and stress had not been investigated. Our transcriptomics data had shown that *teneurin* was relatively highly expressed in the SRO heart compared to the other tissues (16), which led to this study.

The spatial distribution of TCAP in the SRO heart was analyzed using IHC, demonstrating that TCAP is mainly localized in the atria with lower abundance in the ventricle. TCAP was localized to several areas of heart epithelia in both the atria and the ventricle, and in some regions of the atria, ir-TCAP was interspersed from the peripheral epithelial tissues to the inner walls of the heart chambers. GABA, tyrosine hydroxylase and FMRF-amide are recognized neural markers (45–47), and these were used in this study to observe the distribution of neurons in the heart. The distribution of TCAP immunoreactivity followed that of the neuronal markers. GABA and tyrosine hydroxylase were present in the peripheral epithelial cells, and GABA and FMRF were localized in neurons within the cardiac tissue, whereas TCAP was found in both peripheral epithelial and inner heart chambers. This demonstrates that TCAP occurs in the general region as the neural markers in the SRO heart and that teneurin/TCAP is likely to be produced in neuronal tissue rather than cardiomyocytes.

To further explore the role of TCAP in the heart, we performed a pull-down assay to identify potential binding partners and interactions. The pull-down assay showed that sroTCAP binds to Ec-SOD (two different isoforms) and GAPDH in the heart, as Abramov, Suwansa-ard (16) previously reported in hemocytes. In SRO hemocytes, TCAP-GAPDH binding was proposed to be involved in apoptosis inhibition under oxidative stress (16), alluding that this pathway may also be present in the SRO heart. Twenty-five additional proteins were also detected as potential binding partners, suggesting that compared to hemocytes, the heart expresses a different set of proteins with different functions that can interact with sroTCAP, and together with the use of a more sensitive mass spectrometer (TripleTOF^®^ 6600+), low abundance binding proteins have been detected. Several proteins involved in energy metabolism, protein chaperones and structural proteins were also identified in the pull-down assay, suggesting they may interact with sroTCAP or are accessory proteins that bind to the binding partners of sroTCAP. Metabotropic glutamate receptor 1 (mGluR), detected in one of the pull-down replicates (LC-MS/MS spectra of mGluR peptides are shown in **Figure S2**), was the only receptor detected in this assay. Interestingly, mGluR was shown to be involved in the central control of cardiac activity in mammals (48, 49), consistent with its detection in the oyster heart. Evaluation of the mGluR expression in the SRO hearts showed a significant upregulation in the SS group compared to ST (*P =* 0.0109), AT (*P =* 0.0109*)*, and AS (*P =* 0.0078) (**Figure S3**). This shows a stress specific response of mGluR expression in the oyster heart which was modulated by TCAP administration under the same stress condition. The mGluRs are an important family of G-protein-coupled receptors in neural tissue and are involved in the modulation of synapse signalling, neuronal excitability and regulation (49). The activation of mGluRs promotes neuronal growth and prevents neural degeneration under conditions of cellular stresses, including the modulation of nitric oxide oxidative damage and apoptosis induction (49–51), similar to the effects observed following TCAP administration onto murine neuronal cells (26, 32, 52). Evidence in vertebrates shows that TCAP interacts with latrophilin receptors and may be an endogenous ligand (53–55). Latrophilins 1 and 2 have been identified in SRO and have a high expression in the heart compared to the other tissue (**Figure S4**) However, latrophilins were not detected in the pull-down assay using SRO heart proteins, though their interaction cannot be excluded. It remains to be determined whether TCAP could be the ligand for mGluR in invertebrates.

Previous studies predicted that TCAP might be intrinsically unstructured and able to change its conformation as it lacks a well-defined 3D structure (16, 56, 57). In its cleaved or uncleaved form, TCAP may be able to unfold, leading to diverse protein conformations (56, 57), a characteristic of moonlighting proteins (58, 59). This is consistent with our observation that sroTCAP can interact with multiple proteins and protein families in the SRO heart.

Given that sroTCAP appears to interact with a diverse range of SRO proteins and is also present in the heart, a comparative transcriptome analysis was performed to further understand the molecular changes induced by sroTCAP administration under stress conditions. In the heart, only a small proportion (out of all identified transcripts) were considered significantly DEG between the treatment groups, with sroTCAP administration under stress compared to sroTCAP at ambient group showing the largest difference. This suggests that sroTCAP stimulated extensive transcriptional changes during stress and ambient conditions, emphasizing that the effects of sroTCAP on SRO are significantly different when delivered under different conditions.

As expected, stress alone showed a response at the gene expression level, and enriched GO term analysis indicated that several biological functions were shared among stress treatments, regardless of sroTCAP administration. These included ‘gamma aminobutyric acid biosynthetic processes’, ‘response to stimuli’, ‘signalling’, ‘protein folding’, and ‘regulation of transcription from RNA polymerase II promoter’, which were considered stress-induced. However, when sroTCAP was administered to stressed or ambient control oysters, we found that the ‘apoptotic process’ was upregulated under stress control but downregulated under stress with sroTCAP, indicating that sroTCAP can modulate apoptosis under stress, as previously reported in a mouse hypothalamic cell line (N38) (26) and SRO hemocytes (16). ‘Reactive oxygen species metabolic process’ was downregulated following sroTCAP administration at ambient compared to ambient control (AT *vs* AS), demonstrating that sroTCAP may modulate ROS abundance in cardiac tissue under ambient conditions. These results are consistent with previous studies, which showed that TCAP could reduce ROS abundance under ambient and stress conditions and modulate apoptosis in response to stress in SRO hemocytes and a mouse hypothalamic cell line (16, 26).

sroTCAP administration did not change *teneurin* expression in SRO hemocytes under ambient or stress conditions (16). However, in the SRO heart, sroTCAP significantly increased teneurin expression under stress compared to ambient and stress controls. A similar result was observed with rainbow trout (*Oncorhynchus mykiss*) TCAP-3 which was shown to modulate the expression of its precursor teneurin, where 1 nM attenuated expression and 100 nM increased expression *in vitro* (60). Vysokov, Silva (61) also found that HEK293 cells overexpressing teneurin-2 showed overall decreased teneurin expression, likely due to increased dissociation of its intracellular domain. This intracellular domain can translocate to the nucleus, where it can affect teneurin expression and other genes (62, 63). The liberated TCAP may bind to teneurins and trigger its intracellular domain to regulate a suite of genes that may control the stress response and could explain why teneurin expression was significantly higher in the oyster group administered with sroTCAP under stress. A positive feedback mechanism may ensue, where increased teneurin expression leads to the generation of more TCAP, which in turn increases the stress-mitigating capacity of teneurin/TCAP.

Several key genes related to heart function and stress were differentially expressed among the treatment groups. Downregulation of *myosin* expression was observed only in comparison groups having sroTCAP injection under stress (ST *vs* AS, ST *vs* AT and ST *vs* SS) but not in ambient TCAP (AT *vs* AS) and stress control (SS *vs* AS). In rats, TCAP-1 administration upregulated the *myosin heavy chain* expression in skeletal muscle and remodelled the muscle tissue (64). Specifically, short-term (5 days of daily administration) exposure to TCAP-1 changed the muscle fibre type toward slow-twitch fibres, leading to a significant change in function (64). The reduction in *myosin* and *myosin heavy chain* expression in the SRO may demonstrate that sroTCAP can remodel the heart muscle fibre type, changing its activity in response to different abiotic conditions (i.e. temperature, salinity).

Of the investigated calcium channels in cardiomyocytes, which include *ryanodine receptor (RYR)*, *L-type Ca2+ channel* and *stromal interaction molecule 1 (STIM1)*, *IP3R* was the only DEG detected when sroTCAP was delivered during stress compared to ambient conditions. IP3Rs are cation channels that facilitate Ca^2+^ release in cardiomyocytes and the delivery of Ca^2+^ to the mitochondria (65, 66). The administration of mouse TCAP-1 induced cytosolic Ca^2+^ influx in mouse C2C12 myoblast cells, which were inhibited using IP3R blockers (67). Chronic activation of IP3R led to its downregulation in mice oocytes (68), and compounds triggering the production of the ligand inositol 1,4,5-trisphosphate (IP3) downregulated *IP3R* (69). This implies that sroTCAP can stimulate IP3R either directly or indirectly by inducing IP3 production. Furthermore, TCAP-1 influenced rat skeletal muscle contractile kinetics *via* Ca^2+^ dynamic and changed the muscle contraction, including increased peak twitch force and slower contraction velocity (67). Changes to *IP3R* could affect the frequency and duration of Ca^2+^ release (68), and in the SRO heart, it would affect the contractile function of cardiomyocytes.

In the SRO, HSPs, antioxidants, apoptosis and osmoregulation genes are known to associate with cellular stress response (15, 16) and were found to be differentially expressed between the different treatment groups in this study. Antioxidants play an important role in cells to balance the reactive oxygen/nitrogen species and prevent molecular and cellular damage, which leads to disease (2). Differentially expressed antioxidant genes were only detected in groups exposed to sroTCAP under both ambient and stress and were mostly downregulated. However, the heart had a few antioxidant DEGs compared to SRO hemocytes under the same treatments and conditions (16). Several different HSP genes were upregulated in the stress control group; of those, only half were upregulated when sroTCAP was administered to the stress group *vs* the ambient groups. It is interesting to note that while 8 HSPs were differentially expressed in response to stress, the administration of sroTCAP under stress conditions modulated the expression of 4 HSPs and maintained them at level of an unstressed animal. While the other HSPs expression was elevated by stress and was not impacted by TCAP. This suggests that sroTCAP has specificity in modulating the HSP response which allows the upregulation of some HSP genes while modulating others. The modulation of several HSP genes due to sroTCAP administration under stress and ambient conditions had also occurred in SRO hemocytes (16), suggesting sroTCAP’s ability to modulate the cellular stress response to different abiotic conditions *via* the modulation of HSPs. Genes associated with apoptosis were not detected in the stress sroTCAP groups; however, apoptosis inhibitor genes were upregulated in the stress control groups. Previous transcriptome studies on mollusc gill tissue stressed with low salinity altered the expression of *baculoviral IAP repeat-containing protein 2* and *inhibitor of apoptosis* genes, which is consistent with our data (70, 71). This implies that under stress, sroTCAP could repress the anti-apoptosis response, which resembles the gene profile of a non-stressed animal. As the stress treatment in this experiment included low salinity, genes responsible for osmoregulation were investigated. The expression of ion channels and transporter genes were predominantly differentially expressed in stress with sroTCAP comparison groups, with most genes being upregulated. This may suggest that sroTCAP could compensate for the low osmolarity and osmotic shock by increasing the expression of ion channels and transporter genes, thereby promoting the uptake of ions and small molecule in the SRO heart. This observation was also noted in SRO hemocytes subjected to the same conditions (16).

Transcription factors are a diverse group of molecules that can react to internal and external stimuli and alter gene expression, which can be induced under stress (72). sroTCAP administration led to a significant change in the expression of several transcription factors that were not differentially expressed in the stress control group, indicating that sroTCAP may act on multiple genes to modulate the stress response and promote animal survival. An example of this is a *putative zinc finger protein* that was significantly upregulated under stress control (SS *vs* AS) but significantly downregulated when sroTCAP was administered under stress (ST *vs* SS). These results indicate that, given the same stress treatment, the gene expression profile of the heart was considerably different when FSSW control or sroTCAP were injected.

The gene expression profile in the stress control (SS *vs* AS) can be seen as an adaptive response to stress, and sroTCAP seems to have a modulating effect under similar stress conditions. For example, several apoptosis inhibitor genes and *HSP 70 B2-like, 20* and *68-like* genes were upregulated under stress but remained unchanged post sroTCAP delivery under the same stress condition. Similarly, a few transcription factors were downregulated under stress but were unchanged in the stress with sroTCAP group.

The molecular data suggest that sroTCAP could broadly affect many physiological processes of the heart but does not yet demonstrate its direct effect on heart function. Therefore, the physiological effects of sroTCAP on the SRO heart were investigated. The effect of synthetic sroTCAP on SRO cardiac contractility was tested using a force transducer, applying two modes of administration: pericardial and IM injection. Both routes of administration resulted in bradycardia. A dose 10-fold lower (1 pmol) was still cardiac bioactive, having similar effects to the 10 pmol sroTCAP. Nonetheless, both delivery methods and concentrations showed that sroTCAP induced transient bradycardia.

Whether a slower heartbeat in oysters correlates to a relaxed state is unknown; however, in the snail *Helix pomatia*, mechanical stress led to a 20% increase in HR and tachycardia-like effects (73). The opposite was observed in SRO heart exposed to sroTCAP, which may indicate that it has a role in modulating HR and stress in molluscs. Other factors have been demonstrated to affect the heart rate of different molluscs, including environmental conditions such as temperature, oxygen, and feed. When these parameters are increased or decreased, the heartbeat increases and decreases respectively, emphasizing the correlation between heart rate, environmental conditions, and metabolism (8–10). In several invertebrate species (*Spisula solidissima*, *Callinectes sapidus*, *Panopeus herbstii* and *Busycotypus canaliculatus*), hypoxia or anoxia consistently induced bradycardia (74). Koester, Dieringer (9) reported that bradycardia in response to low oxygen and low temperatures is thought to be a mechanism for energy conservation. A correlation between sroTCAP and energy conservation and stress coping mechanisms was described in SRO hemocytes (16).

Changes to the HR in response to temperature alteration are thought to occur *via* thermoreceptors on the mantle (75). However, sroTCAP seemingly operates under a different mechanism. mGluR was found to interact with sroTCAP in the pull-down assay, and the transcriptome data suggest that sroTCAP triggered the IP3R, both of which have a role in cardiac activity (48, 49, 65, 66). Molluscan heart rhythm, like higher animals, is modulated by neurons, nerves, and ganglia (76). Although the visceral ganglion has protruding nerves that innervate the heart to affect heart contractions (77), it is spatially located distant from the heart. We therefore suggest that sroTCAP most likely targets the proximal nerve tissue. This is supported by the fact that pericardial delivery altered the heartbeat independent of direct contact with the visceral ganglia and suggests that the response was through nerve tissue around the heart (i.e. cardiac ganglion) or the neurons within the heart.

## 5 Conclusions

For the first time, TCAP was investigated for its effects on SRO cardiac tissue. Our findings show that TCAP may be localized to the neural innervation network of the heart, with a similar distribution pattern to neuronal markers such as GABA, tyrosine hydroxylase and FMRFamide. mGluR may be a potential receptor for sroTCAP in the SRO heart based on protein-protein interactions and requires further investigations. Several other proteins could be partnered with sroTCAP intracellularly, enforcing the presumed intrinsic unstructured property of TCAP. Comparative transcriptome analysis showed that sroTCAP regulates the expression of *myosin* and *IP3R* genes, which could remodel the heart under different environmental conditions and affect the contractile function of cardiomyocytes, respectively. Furthermore, sroTCAP led to the modulation of HSPs, apoptosis response, and osmoregulation genes but only affected a small range of genes related to the antioxidant stress response. The administration of 10 and 1 pmol sroTCAP significantly lowered the SRO heart BPM and changed the heart rhythm, causing a temporary bradycardia-like effect associated with energy conservation and stress mitigation. Overall, this study demonstrated that sroTCAP is cardiac bioactive, interacts with endogenous proteins, modulates several genes under ambient and stress conditions, and causes significant changes to heart rhythm in the SRO. We report an array of stress mitigating effects of TCAP in molluscan hearts, which should be investigated in higher animals, as the reported effects could potentially have meaningful therapeutic benefits on veterinary and human conditions and diseases.

## Data availability statement

The datasets presented in this study can be found in online repositories. The transcriptome data presented in the study are deposited in the NCBI SRA database, accession number PRJNA804582 (https://www.ncbi.nlm.nih.gov/bioproject/PRJNA804582/) and the proteomics data are deposited in the ProteomeXchange Consortium, accession number PXD039155 (https://www.ebi.ac.uk/pride/archive/projects/PXD039155).

## Author contributions

AE, LP, and DL conceptualized the manuscript. TA, SS-A, PD, FR, SC, DL, and AE contributed to the design of the manuscript. TA, SS-A, and PD conducted the experiments. TW contributed to the LC-MS/MS analysis. TA, SS-A, SC, and AE wrote the manuscript. MD and WO’C provided the experimental animals. All authors contributed to the article and approved the submitted version.
