# Supplementary material for: A novel role for Teneurin C-terminal Associated Peptide (TCAP) in the regulation of cardiac activity in the Sydney rock oyster, Saccostrea glomerata
Source: Front Endocrinol (Lausanne). 2023 Feb 6;14:1020368. doi: 10.3389/fendo.2023.1020368 (PMC9939839; doi:10.3389/fendo.2023.1020368)
Supplement: Supplementary file 1 [file DataSheet_1.docx]

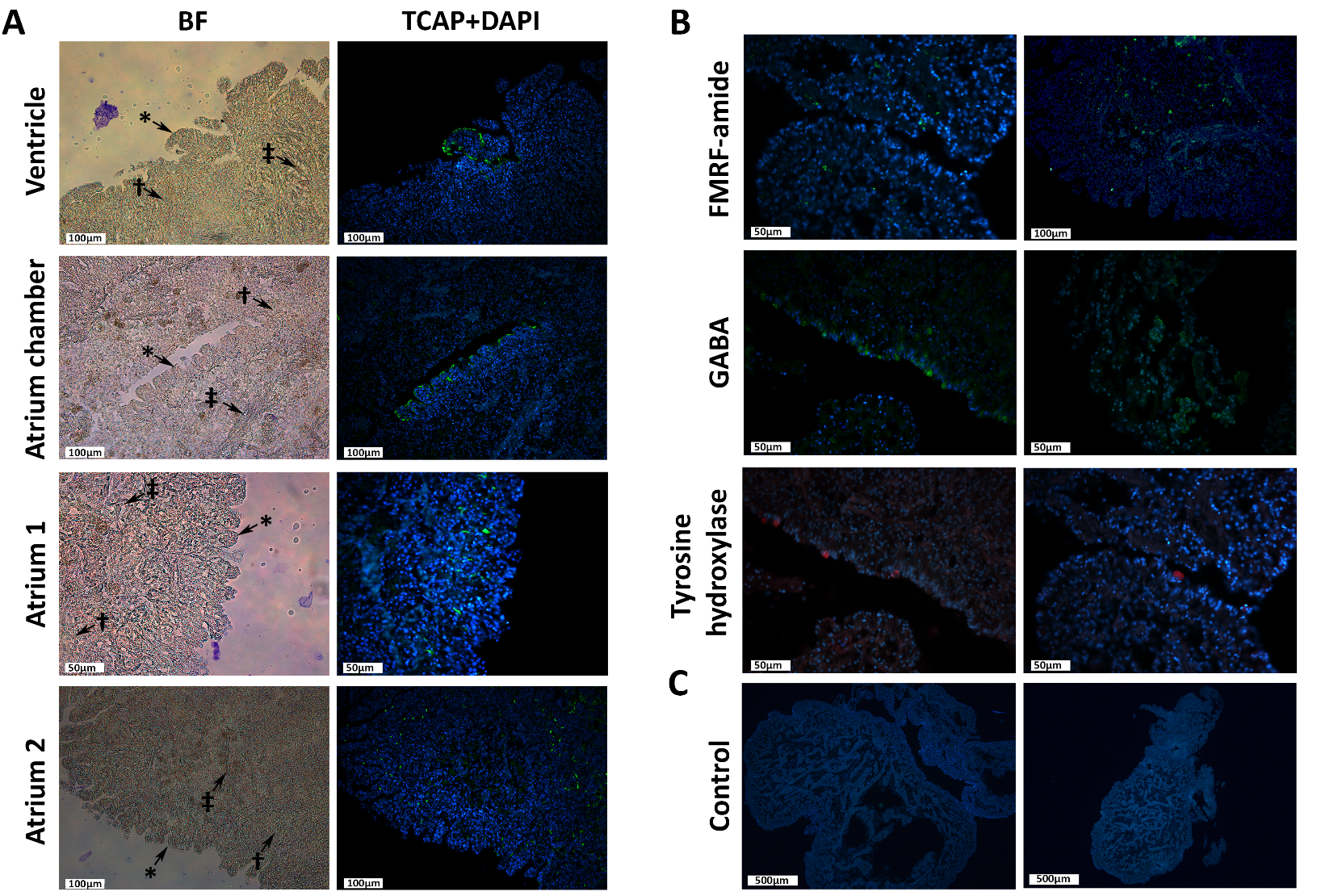
**Figure S1.** Immunohistochemistry of fixed SRO heart sections(ventricle and atria) showing (**A**) immunoreactivity using anti-TCAP antibodies (green) and (**B**) using anti-FMRF-amide, anti-GABA (green), and tyrosine hydroxylase (red). (**C**) Negative controls, secondary antibodies only. BF- bright field; DAPI stain shows cell nucleus in blue. Black arrows point to * epithelial cells, † connective tissue, ‡ muscle fibres. Scale bars 50 and 100 µm.

**Table S1.** Proteins identified from LC-MS/MS (QTOF X500R mass spectrometer) of pull-down assay using SRO heart lysate and biotinylated sroTCAP. -10lgP value represents the confidence of protein identification (cut-off value >20), Coverage represents the detected fragment coverage of the full protein, #Peptides represent the total number of peptide hits per protein, and #Unique represents the number of unique peptides detected per protein. *, also identified in Abramov, Suwansa-ard (16).

| **Gene ID** | **Protein annotation** | **-10lgP** | **Coverage (%)** | **#Peptides** | **#Unique** |
| --- | --- | --- | --- | --- | --- |
| Sgl003210 | putative aminopeptidase W07G4.4 | 210.9 | 35 | 17 | 17 |
| Sgl003905 | glyceraldehyde 3-phosphate dehydrogenase* | 188.19 | 52 | 14 | 14 |
| Sgl023942 | extracellular superoxide dismutase [Cu-Zn]-like | 182.44 | 25 | 11 | 9 |
| Sgl009446 | complement C1q-like protein 4 | 150.44 | 25 | 5 | 5 |
| Sgl018292 | cytosolic malate dehydrogenase | 147.19 | 24 | 5 | 5 |
| Sgl004322 | carbonic anhydrase-like | 144.86 | 23 | 4 | 4 |
| Sgl023943 | extracellular superoxide dismutase [Cu-Zn]-like* | 119.94 | 18 | 4 | 2 |
| Sgl001248 | ependymin-related protein 1 | 53.27 | 18 | 1 | 1 |
| Sgl024699 | citrate synthase, mitochondrial-like | 52.32 | 4 | 2 | 1 |
| Sgl018613 | heat shock protein 20 | 49.68 | 15 | 2 | 2 |
| Sgl009849 | AAAChain AAA, Streptavidin | 36.2 | 11 | 1 | 1 |

**Table S2.** Background proteins identified from LC-MS/MS of the negative control pull-down assay using SRO heart lysate without biotinylated sroTCAP. These proteins were subtracted from proteins identified in the pull-down assay using biotinylated sroTCAP. Gene ID* from the online database of Sydney Rock Oyster Genome (http://soft.bioinfo-minzhao.org/srog/index.html). -10lgP value represents the confidence of protein identification (cut-off value >20), Coverage represents the detected fragment coverage of the full protein, #Peptides represent the total number of peptide hits per protein, and #Unique represents the number of unique peptides detected per protein.

| **Gene ID*** | **Protein annotation** | **-10lgP** | **Coverage (%)** | **#Peptides** | **#Unique** |
| --- | --- | --- | --- | --- | --- |
| Sgl012534 | ATP synthase subunit beta, mitochondrial | 219.29 | 50 | 17 | 17 |
| Sgl003537 | cathepsin B-like | 197.57 | 42 | 15 | 15 |
| Sgl018022 | tropomyosin isoform X5 | 160.22 | 45 | 15 | 15 |
| Sgl009147 | ATP synthase subunit alpha, mitochondrial-like | 128.61 | 18 | 4 | 4 |
| Sgl013322 | uncharacterized protein LOC105332169 isoform X2 | 127.98 | 11 | 7 | 7 |
| Sgl008227 | radixin-like isoform X4 | 118.97 | 8 | 4 | 4 |
| Sgl014431 | elongation factor 1 alpha | 112.64 | 18 | 5 | 5 |
| Sgl019516 | actin-related protein 2/3 complex subunit 3-like | 106.16 | 25 | 3 | 3 |
| Sgl016380 | aldehyde dehydrogenase, mitochondrial-like | 103.84 | 9 | 3 | 3 |
| Sgl014168 | 40S ribosomal protein SA | 102.36 | 22 | 4 | 4 |
| Sgl007029 | Protein CBG29119 | 101.3 | 62 | 4 | 3 |
| Sgl007519 | 60S ribosomal protein L12-like | 96.18 | 34 | 4 | 4 |
| Sgl019241 | actin-related protein 3 isoform X2 | 90.29 | 7 | 3 | 3 |
| Sgl005002 | 60S acidic ribosomal protein P0-like | 88.57 | 11 | 3 | 3 |
| Sgl015119 | 40S ribosomal protein S3 | 79.5 | 9 | 2 | 2 |
| Sgl023302 | putative uncharacterized oxidoreductase YDR541C | 76.13 | 10 | 3 | 3 |
| Sgl001948 | 60S ribosomal protein L6 | 74.11 | 11 | 2 | 2 |
| Sgl016776 | serine/threonine-protein phosphatase alpha-2 isoform | 73.03 | 9 | 2 | 2 |
| Sgl014768 | heat shock protein 70 | 70.75 | 4 | 2 | 2 |
| Sgl008177 | actin-related protein 2/3 complex subunit 5-like | 67.67 | 12 | 2 | 2 |
| Sgl016464 | filamin-C-like isoform X1 | 67.46 | 1 | 1 | 1 |
| Sgl000068 | Hypothetical predicted protein | 66.9 | 1 | 2 | 2 |
| Sgl022647 | complement C1q-like protein 4 | 66.52 | 15 | 1 | 1 |
| Sgl003412 | 14-3-3-like protein 2 isoform X1 | 64.99 | 10 | 2 | 1 |
| Sgl003413 | 14-3-3 protein | 64.99 | 8 | 2 | 1 |
| Sgl005300 | actin-3 isoform X4 | 64.77 | 4 | 2 | 1 |
| Sgl008307 | 40S ribosomal protein S16 | 62.17 | 17 | 2 | 2 |
| Sgl014033 | multifunctional protein ADE2-like | 60.86 | 7 | 2 | 2 |
| Sgl001565 | hypothetical protein FL81_09400 | 60.48 | 36 | 2 | 1 |
| Sgl007879 | retrograde protein of 51 kDa-like isoform X4 | 57.65 | 3 | 1 | 1 |
| Sgl005615 | 40S ribosomal protein S10-like | 57.3 | 15 | 2 | 2 |
| Sgl018575 | THO complex subunit 4-like | 56.47 | 5 | 1 | 1 |
| Sgl008020 | 16 kDa calcium-binding protein | 54 | 5 | 1 | 1 |
| Sgl025972 | kelch-like protein 12 | 53.8 | 1 | 1 | 1 |
| Sgl021900 | actin-related protein 2/3 complex subunit 4 | 52.54 | 30 | 2 | 2 |
| Sgl009169 | 40S ribosomal protein S15-like | 52.48 | 15 | 1 | 1 |
| Sgl008849 | suppressor of tumorigenicity 14 protein homolog | 49.47 | 3 | 1 | 1 |
| Sgl023922 | stress-70 protein, mitochondrial-like | 49.37 | 2 | 1 | 1 |
| Sgl012099 | stress-70 protein, mitochondrial-like | 49.37 | 2 | 1 | 1 |
| Sgl021656 | histone H2A | 47.61 | 7 | 1 | 1 |
| Sgl018583 | histone H2A.V | 47.61 | 7 | 1 | 1 |
| Sgl010038 | histone H2A | 47.61 | 7 | 1 | 1 |
| Sgl011845 | histone H2A-like | 47.61 | 7 | 1 | 1 |
| Sgl015490 | histone H2A | 47.61 | 5 | 1 | 1 |
| Sgl018580 | Histone H2A.v | 47.61 | 4 | 1 | 1 |
| Sgl012162 | 78 kDa glucose-regulated protein | 47.08 | 3 | 1 | 1 |
| Sgl006224 | ATP synthase subunit O, mitochondrial | 45.88 | 5 | 1 | 1 |
| Sgl004383 | ubiquitin-like protein FUBI | 43.88 | 9 | 1 | 1 |
| Sgl018652 | Alpha-crystallin B chain | 42.64 | 11 | 1 | 1 |
| Sgl000344 | small nuclear ribonucleoprotein Sm D3-like | 42 | 8 | 1 | 1 |
| Sgl013834 | heterogeneous nuclear ribonucleoprotein R-like isoform X1 | 38.58 | 2 | 1 | 1 |
| Sgl013323 | uncharacterized protein LOC117681453 | 36.5 | 6 | 1 | 1 |
| Sgl005231 | 40S ribosomal protein S4-like | 35.06 | 4 | 1 | 1 |
| Sgl015111 | 60S ribosomal protein L28-like | 33.95 | 8 | 1 | 1 |
| Sgl017844 | 14-3-3 zeta | 33.25 | 9 | 2 | 1 |
| Sgl012284 | eukaryotic peptide chain release factor subunit 1 | 32.52 | 3 | 1 | 1 |
| Sgl006322 | eukaryotic translation initiation factor 2A-like | 32.08 | 2 | 1 | 1 |
| Sgl008775 | 40S ribosomal protein S25-like | 28.63 | 11 | 1 | 1 |
| Sgl002533 | Hypothetical predicted protein | 28.22 | 5 | 1 | 1 |
| Sgl016176 | proteasome subunit beta type-2-like | 23.41 | 4 | 1 | 1 |
| Sgl004006 | galactosylceramide sulfotransferase-like | 23.4 | 1 | 1 | 1 |
| Sgl023639 | fasciclin-1-like isoform X2 | 23.4 | 1 | 1 | 1 |
| Sgl007243 | cytoplasmic dynein 1 heavy chain 1 isoform X9 | 23.07 | 0 | 1 | 1 |


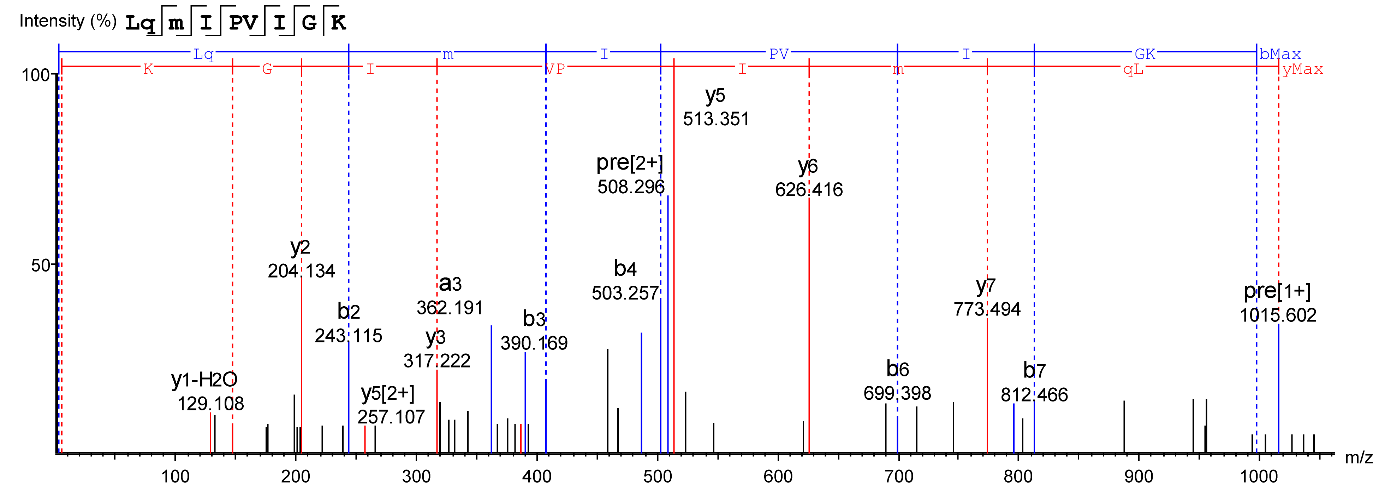


**Figure S2.** Spectra of the mGluR (Sgl025823) peptides detected in the pull dopwn assay in SRO heart. Two identical peptides of mGluR were detected.

**Figure S3**. Relative expression (RPKM) of the Metabotropic glutamate receptor 1 gene (mGluR), in the heart of SRO under ambient or stress conditions and IM-injected with 5 pmol sroTCAP or FSSW (n=3). AS- oysters at ambient condition injected with FSSW, AT- oysters at ambient condition injected with sroTCAP, SS- oysters under stress condition injected with FSSW and ST- oysters under stress condition injected with sroTCAP. **P* ≤ 0.05, ** *P* ≤0.01.

**Table S5**. Data for heart rate in bpm after delivery of FSSW or sroTCAP, mean±SEM. ****, P<0.0001. ND, not determined.

|  | 1 pmol | | 10 pmol | |
| --- | --- | --- | --- | --- |
|  | Control | sroTCAP | Control | sroTCAP |
| Pericardial | 12.7 ± 0.46**** | 6.5 ± 0.9**** | 13.9 ± 0.4 | 5.1 ± 0.08**** |
| Intramuscular | ND | ND | 13.05 ± 0.41 | 6.02 ± 0.3**** |


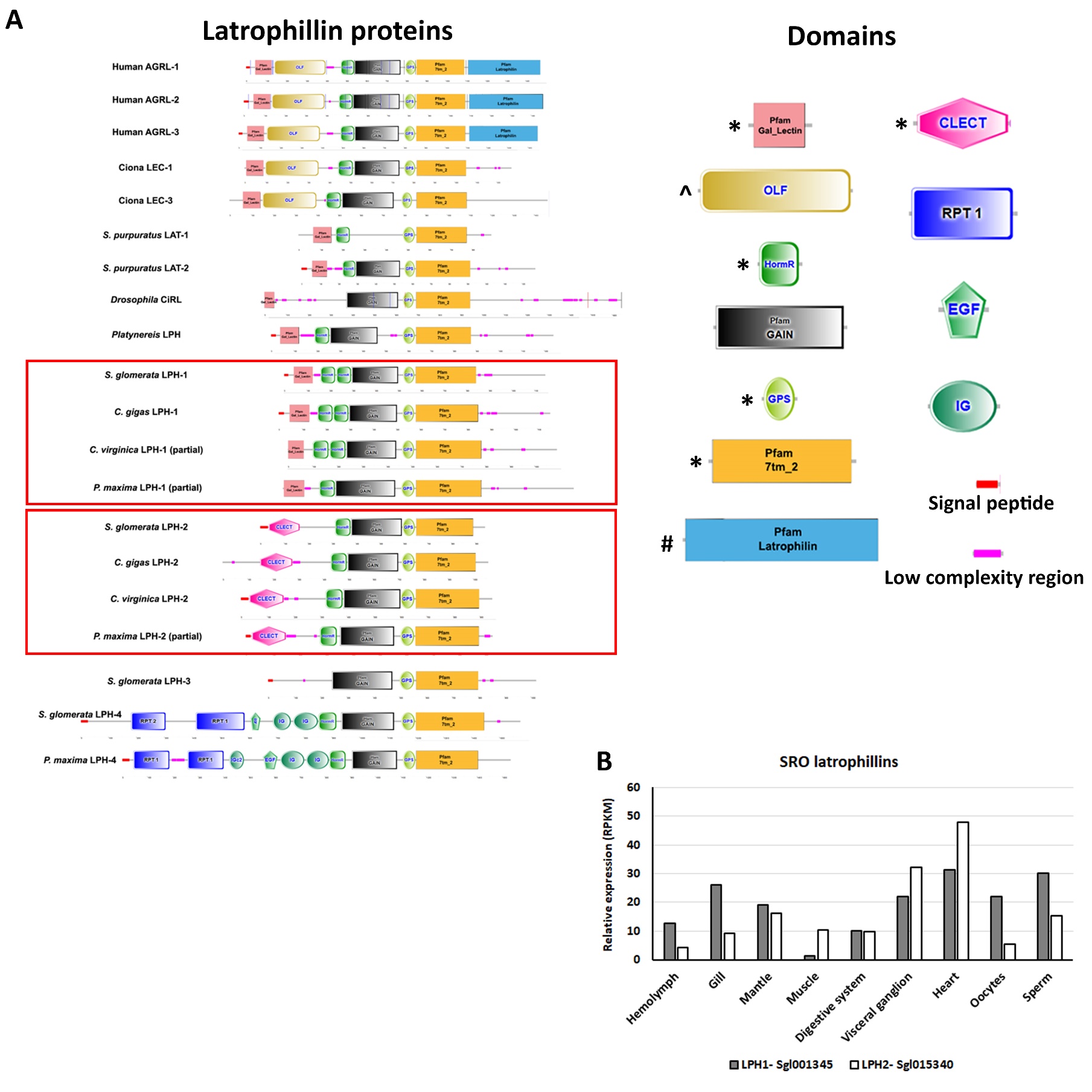
**Figure S4.** Identification of latrophilins in SRO and relative expression in tissues. **(A)** Latrophilin protein comparison of vertebrates and invertebrates, obtained from NCBI database (https://www.ncbi.nlm.nih.gov/), showing PFAM domains (SMART- <http://smart.embl-heidelberg.de/>). * domains common in vertebrate and invertebrate latrrophilins, # domains specific to chordate latrophilins, ^ domains specific to protochordates/chordates. Only SRO latrophilin 1 and 2 (LPH 1-2, red boxes) had domains conserved with known latrophillins. Putative SRO latrophilin 3 and 4 lack key domains and/or have domains not found in known latrophilins, therefore they are not considered latrophilins. **(B)** Relative expression (in RPKM) of LPH 1 and 2 in SRO tissues showing highest expression in the heart followed by the visceral ganglion.
